# Plant and Animal-Based Dietary Patterns and Cardiometabolic Diseases in the Brazilian Population: Cross-Sectional Analysis of the Brazilian National Health Survey

**DOI:** 10.3390/nu17213448

**Published:** 2025-10-31

**Authors:** Poliana E. Correia, Lauren Bisi, Minghui Zhang, Yunxiang Sun, Bárbara B. Martins, Olavo S. C. Porepp, Veronica Colpani, Laura B. Kunzler, Paula P. Teixeira, Gabriel T. Ferrari, Lenita Zajdenverg, Elisa Brietzke, Mariana P. Socal, Fernando Gerchman

**Affiliations:** 1 Division of Endocrinology and Metabolism, Hospital de Clínicas de Porto Alegre, Porto Alegre 90035-903, RS, Brazilvecolpani@gmail.com (V.C.); fgerchman@hcpa.edu.br (F.G.); 2 Postgraduate Program in Medical Sciences—Endocrinology, Department of Internal Medicine, Faculdade de Medicina, Universidade Federal do Rio Grande do Sul (UFRGS), Porto Alegre 90035-003, RS, Brazil; 3 Johns Hopkins Bloomberg School of Public Health, Baltimore, MD 21205, USA; 4 Department of Epidemiology, Johns Hopkins Bloomberg School of Public Health, Baltimore, MD 21205, USA; f006zjg@dartmouth.edu; 5 Núcleo de Avaliação de Tecnologias em Saúde, Hospital Sírio-Libanês (HSL), São Paulo 01431-000, SP, Brazil; 6 Department of Nutrition, Faculty of Medicine, Universidade Federal do Rio Grande do Sul, Porto Alegre 90035-003, RS, Brazil; gtferrari@hcpa.edu.br; 7 Internal Medicine Department, Federal University of Rio de Janeiro, Rio de Janeiro 21941-853, RJ, Brazil; 8 Center for Neuroscience Studies (CNS), Department of Psychiatry, Queen’s University School of Medicine, Kingston, ON K7L 3N6, Canada; 9 Department of Health Policy and Management, Johns Hopkins Bloomberg School of Public Health, Baltimore, MD 21205, USA

**Keywords:** plant-based diet, animal-based diet, public health nutrition, National Health Survey, dietary patterns

## Abstract

**Background:** Brazil’s dietary patterns and significant socioeconomic and geographic diversity present unique challenges for the prevention of cardiometabolic diseases. **Methods:** In this cross-sectional study, we analyzed data from a nationwide representative survey to understand how dietary patterns related to cardiometabolic diseases. We classified the dietary pattern of participants as whole plant-based, processed plant-based, and animal-based. Then, they were categorized into high, intermediate, and low consumption. Logistic regression analysis was used to test the prevalence of obesity, hypertension, hypercholesterolemia, diabetes, stroke, and heart diseases according to the level of intake of each of the three dietary patterns. **Results**: Compared to the low intake of a whole plant-based dietary pattern, a high intake was associated with a lower prevalence of obesity (OR 0.64; 95% CI 0.54, 0.75) and hypercholesterolemia (OR 0.69, 95% CI 0.56, 0.85). A processed plant-based dietary pattern (including items such as soda and sweets) was inversely associated with the prevalence of obesity (OR 0.90; 95% CI 0.83, 0.97), hypertension (OR 0.82; 95% CI 0.76, 0.88), hypercholesterolemia (OR 0.81; 95% CI 0.74, 0.88), and diabetes (OR 0.53; 95% CI 0.48, 0.59). A high intake of animal-based dietary patterns was associated with a lower prevalence of heart diseases (OR: 0.60; 95% CI 0.40, 0.90). **Conclusions**: In this cross-sectional analysis, greater adherence to specific dietary patterns was associated with differences in the prevalence of cardiometabolic conditions. However, causality cannot be established, and longitudinal studies are warranted to confirm these findings.

## 1. Introduction

Previous evidence suggests that the growing prevalence of noncommunicable diseases (NCDs) and related deaths is partly driven by an imbalance characterized by the excessive consumption of refined carbohydrates, sugar-sweetened beverages, added salt, unhealthy fats, and ultra-processed foods, alongside the insufficient intake of vegetables, fruits, unsaturated fats, and whole grains [1]. Although most of these deaths are especially concentrated in low to middle-income countries, it is less clear how dietary patterns influence the development of NCDs in these countries, especially in South America. Brazil, the largest and most populous country in South America, possesses a rich diversity of food sources shaped by the vast array of leaves, roots, fruits, nuts, and fish found across diverse biomes, including extensive coastal, lacustrine, riverine, and rainforest ecosystems. Additionally, Brazil hosts one of the world’s largest animal protein industries, spanning beef, pork, and poultry—particularly concentrated in the South and Southeast regions of the country [2]. All these factors, allied with regional, ethnic, and cultural values, may define the alimentary habits of Brazilians and be a major determinant of the impact of NCDs in this population.

Plant-based is a dietary pattern that the EAT-Lancet Commission on Food, Planet, and Health brought together in a proposal elaborated by experts and world governmental leaders, with the potential of averting about 11 million deaths per year [3]. The definition of a plant-based diet varies between studies, but a whole plant-based diet is usually defined as a dietary pattern that emphasizes the primary consumption of plant-derived foods in their whole, unprocessed form, such as vegetables, fruits, grains, and nuts, while minimizing or eliminating the intake of processed foods and refined grains, and limiting but not necessarily excluding the intake of unprocessed animal-based products [3,4].

Recent evidence suggests that the quality of diet within plant-based patterns is important. In large European cohorts, a higher level of adherence to a healthful plant-based index (hPDI) was associated with lower CVD risk, whereas unhealthful patterns (uPDI) were associated with higher risk [5]. Similar findings were observed in the UK Biobank among individuals with prediabetes and diabetes [6]. Replacing modest amounts of red and processed meat with vegetables, legumes, or whole grains is associated with lower cardiovascular risk [7]. Evidence from Australasia shows that this dietary pattern is associated with a lower mortality in subjects with type 2 diabetes mellitus [8]. Although evidence for plant-based patterns has grown, the relationship between meat consumption and cardiometabolic disease remains debated: different studies found that reducing red/processed meat has a small impact on reducing health outcomes and cardiovascular mortality [9,10]. An updated systematic review and meta-analysis, as well as a Burden of Proof study, rated the association between unprocessed red meat consumption and the risk of ischemic heart disease and type 2 diabetes as weak [11,12]. Taken together, these findings suggest that not all plant-based dietary patterns confer the same benefits, underscoring the importance of distinguishing between whole and refined plant components when assessing associations with cardiometabolic diseases.

In Brazil, longitudinal evidence highlights the role of diet quality in cardiometabolic health. In the ELSA-Brazil cohort, the higher consumption of ultra-processed foods (NOVA classification) was prospectively associated with a higher incidence of hypertension and type 2 diabetes, with sugar-sweetened beverages and processed meats standing out among the contributors [13,14]. Complementing these findings, the CUME cohort reported that higher adherence to a healthy plant-based index (hPDI) predicted a lower incidence of hypertension over follow-up, particularly among adults with overweight or lower physical activity [15]. Furthermore, Brazilian analyses applying the EAT-Lancet Planetary Health Diet reported more favorable cardiometabolic profiles with higher adherence, reinforcing the importance of distinguishing minimally processed plant-based foods from refined/processed items in local dietary patterns [16]

Due to the potential impact of the plant-based diet on NCDs, there is a need to evaluate and understand its effect on different populations, not only those with distinct nutritional habits. This is also because the relationship between the diet and development of NCDs may be variable in populations with diverse ethnic and genetic backgrounds, such as Brazilians, among the largest consumers of animal-based food in the world [10,17,18]. Together, these Brazilian studies provide local context and support our emphasis on diet quality and composition patterns. Therefore, this cross-sectional study aimed to examine the associations between three dietary patterns—whole plant-based, processed plant-based, and animal-based—and the prevalence of obesity, hypertension, diabetes, hypercholesterolemia, stroke, and heart disease in a nationally representative sample of Brazilian adults.

## 2. Materials and Methods

### 2.1. Data and Sample

This study used data from the 2019 Brazilian National Health Survey (Pesquisa Nacional de Saúde, PNS), a nationwide cross-sectional population-based household survey, in which individuals answered questions about noncommunicable diseases (NCDs), lifestyle (e.g., physical activity, tobacco use, alcohol intake), and dietary habits. The survey was developed by the Brazilian Institute of Geography and Statistics (Instituto Brasileiro de Geografia e Estatística, IBGE) in partnership with the Ministry of Health. Survey weights were applied to all analyses to account for the complex sampling design and to make representative estimates of the national non-institutionalized population of Brazil aged 15 years and older [19].

A total of 87,678 out of 90,846 PNS respondents who were at least 18 years old and had weight and height data were included in this analysis. In the PNS, the responder had the option of answering the questions with “not applicable” or having the right to not answer the question (“ignored”), which explains the presence of missing data for some variables.

### 2.2. Dietary Patterns

The survey included a questionnaire about health and lifestyle habits, including self-reported weight and height. Participants reported their frequency of food consumption through the Food Frequency Questionnaire (FFQ) by saying how many days (0 to 7) in the past week they consumed different foods: beans, raw and cooked vegetables, fruits, natural fruit juice, milk, sugar-sweetened beverages (soda and artificial fruit juice), fish, red meat, chicken, sweets (cakes or pies, chocolates, candies, cookies or sweet biscuits), and how many days in the last week they substituted lunch or dinner for sandwiches, snacks or pizzas. Based on these data, we then categorized food groups as follows: whole plant-based foods (beans, vegetables, fruits, and natural fruit juice), processed plant-based foods (juice [except sugar-free juice], soda [except sugar-free soda], cookies/sweets, and snacks/prepared meals) and animal-based foods (fish, milk, red meat, and chicken). The processed plant-based group comprises plant-sourced items that have some degree of processing, including processed to ultra-processed products (e.g., sugar-sweetened beverages).

To rank the participants’ whole plant-based food consumption, we divided them among three groups, not mutually exclusive, high, intermediate, and low, according to the frequency participants consumed foods from the whole plant-based food group for 1 week. To rank processed plant-based food consumption, we established only two categories: low and high. To rank the consumption of animal-based foods, we also defined three categories: low, intermediate, and high. For whole plant-based, processed plant-based, and animal-based food groups, the cutoff points were defined using the distribution based on consumption recommendations of each food [20,21]. Each individual food in the sample, therefore, received a separate score for its food consumption in each of the three groups of interest (Table 1).

### 2.3. Main Outcome Measures

The main outcomes examined in this study were the prevalence of obesity, hypertension, diabetes, hypercholesterolemia, stroke, and heart disease. Obesity was defined as a body mass index (BMI) of 30 kg/m^2^ or higher, and it was calculated from the participants’ self-reported weight and height. The presence of each chronic condition was self-reported through the survey question “Has a doctor ever told you that you have (disease name)?” For diabetes and hypertension, we excluded women who reported that they had been diagnosed only during pregnancy.

### 2.4. Main Control Variables

The main covariates considered for adjustment were age, sex assigned at birth, race, marital status, household income, highest education level achieved, urban or rural place of residence, geographical region, physical activity level, smoking status, alcohol intake, overall health status, and dietary pattern (whole plant-based, animal-based, processed plant-based).

Physical activity level included activities practiced during leisure time (e.g., sports), heavy lifting activities in the household or work, and commuting (e.g., walking or riding a bike to school or work). The level of physical activity was categorized as ≥150 min per week or <150 min per week. Tobacco use was categorized as never smoked versus current or former smokers. Alcohol intake frequency was categorized into less than once a month and one or more times a month. All control variables were obtained through self-reported information.

### 2.5. Data Analysis and Statistical Methods

We conducted descriptive analyses using chi-square tests and ANOVA when appropriate to compare groups. Logistic regression analyses were used to test associations between the level of consumption of foods in each of the three dietary groups (whole plant-based, processed plant-based, and animal-based foods) and the prevalence of obesity, hypertension, hypercholesterolemia, diabetes, stroke, and heart diseases. These relationships were tested in unadjusted and adjusted models, controlling for the full set of covariates. All analyses were performed considering the weighting for the complex sample structure in order to represent the Brazilian population, according to the research sample, and the missing data resulted by the “not applicable” and “ignored” options for responders [19].

**Figure A1:**
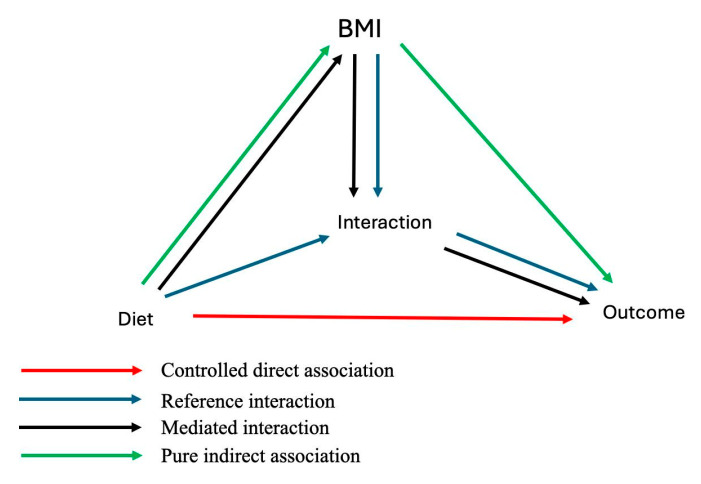
Directed Acyclic Graph (DAG) illustrating the four-way decomposition of the association between dietary patterns and cardiometabolic outcomes, with BMI as a mediator. Red arrow: Controlled direct association—effect of diet on outcome, independent of BMI. Blue arrows: Reference interaction—interaction between diet and BMI affecting the outcome. Black arrows: Mediated interaction—combined effect of diet influencing BMI, which then interacts with diet to influence the outcome. Green arrows: Pure indirect association—mediation pathway where diet influences BMI, which subsequently affects the outcome.

We conducted an exploratory mediation (decomposition) analysis, treating BMI as a potential model on the pathway between dietary patterns and each outcome. Given the cross-sectional design, temporal ordering (exposure → BMI → outcome) cannot be established; therefore, these analyses are descriptive/hypothesis-generating and do not identify causal mediation. Specifically, we examined whether BMI mediated the association between the exposure (low vs. high whole plant-based diet groups) and each outcome, and we also assessed exposure–mediator interaction (BMI × exposure) as a form of effect modification. The mediation analysis decomposed the relationship between dietary patterns and the outcomes of interest into four components: (1) controlled direct association (neither mediation nor interaction between exposure and mediator), (2) pure indirect association (mediation only between exposure and mediator), (3) reference interaction (interaction only between exposure and mediator), and (4) mediated interaction (mediation and interaction between exposure and mediator) (Figure A1). Although a typical mediation analysis requires longitudinal data, we implemented this analysis to test the robustness of our findings—specifically, we wanted to account for the possibility that the association we identified between dietary patterns and the various outcomes of interest had not been spurious and may or may not be related to the presence of obesity. In an effort to check the robustness of our findings, we performed a sensitivity analysis, where we replicated all our analytical models, including the mediation analysis, using data from the prior wave of the PNS survey in 2013.

All analyses were performed using STATA version 14.0 (StataCorp, College Station, TX, USA). The mediation analysis was implemented using the med4way package for STATA. Statistical significance was defined as *p*-value ≤ 0.05. This study followed the EQUATOR STROBE checklist for cross-sectional studies.

### 2.6. Ethical Aspects

The 2019 PNS survey was approved by the Brazilian National Research Ethics Committee/National Health Council as per Opinion No. 3.529.376, issued on 23 August 2019. The present study did not constitute human participant research because only de-identified and publicly available survey results were utilized.

## 3. Results

### 3.1. Demographic Data and Characteristics of the Group

Data from 87,678 participants were analyzed (95.5% of the total 90,846 individuals who responded to the PNS survey). The characteristics of the study sample are shown in Table 2. Mean age was 47.4 (SD = 17.1) years, and 52.48% of the participants were women. Thirty-four percent completed at least primary school, and 77.06% lived in urban areas. Non-smokers were 68.85% of the group, 46.7% had an intake of alcohol at least once a month, and 67.15% reported practicing less than 150 min/week of physical activity. Regarding the self-reported presence of cardiometabolic NCDs, 20.6% had obesity, 8.7% had diabetes, 26.1% had hypertension, 16.6% had hypercholesterolemia, 5.4% had heart diseases, and 2% had stroke (Table S1).

When analyzing data according to the frequency of intake of a whole plant-based dietary pattern, subjects with a high intake were older than 52.05 (SD = 16.72) years. They were predominantly females, white, and had a higher education level compared to those who reported a low and intermediate intake of a plant-based dietary pattern. These subjects were more likely not to drink alcohol and more usually performed equal or more than 150 min/week of physical activity compared with subjects with a lower consumption of whole plant-based foods (Table 2).

### 3.2. Intake of Whole and Processed Plant-Based and Animal Dietary Patterns and Their Relationships with the Prevalence of Diabetes, Hypertension, Heart Disease, Stroke, and Hypercholesterolemia

To examine the relationship between dietary patterns and the prevalence of cardiometabolic diseases, we first conducted univariate analyses (Table S2). When the direction of these results differed from the adjusted estimates obtained from multivariate models, we relied on the latter, as they account for multiple confounders, including sociodemographic, behavioral, and clinical factors. This pattern is consistent with confounding; therefore, adjusted models should be considered primary and crude estimates interpreted cautiously.” We also conducted multiple logistic regression models controlling for the full set of covariates (Table 2) to compare the following groups: (1) low vs. intermediate vs. high frequent intake of whole plant-based dietary pattern, (2) high vs. low frequent intake of a processed plant-based dietary pattern, and (3) high vs. intermediate vs. low frequent intake of an animal-based dietary pattern.

Multivariate analyses showed that, when compared to a low intake of a whole plant-based dietary pattern, high (OR 0.64; 95% CI 0.54, 0.75) and intermediate (OR 0.77; 95% CI 0.68, 0.88) intake levels were associated with a lower prevalence of obesity. A high whole plant-based dietary pattern was also associated with a lower prevalence of hypercholesterolemia compared with a lower one (OR 0.69, 95% CI 0.56, 0.85). A high and intermediate intake of a whole plant-based dietary pattern was not associated with a lower prevalence of diabetes, hypertension, and stroke (Table 2).

Following a processed plant-based dietary pattern was inversely associated with the prevalence of obesity (OR 0.90; 95% CI 0.83, 0.97), hypertension (OR 0.82; 95% CI 0.76, 0.88), hypercholesterolemia (OR 0.81; 95% CI 0.74, 0.88), and diabetes (OR 0.53; 95% CI 0.48, 0.58). There was no association with stroke and heart diseases (Table 2).

In comparison with a low consumption, following a high intake of an animal-based dietary pattern was not associated with the presence of obesity (OR 0.98; 95% CI 0.75, 1.29), hypertension (OR 0.85; 95% CI 0.65, 1.11), hypercholesterolemia (OR 0.92; 95% CI 0.70, 1.22), or diabetes (OR 1.13; 95% CI 0.79, 1.60). However, in comparison to a lower animal-based dietary pattern, a higher one (OR: 0.60; 95% CI 0.40, 0.90) and an intermediate one (OR 0.77; 95% CI 0.60, 0.99) were associated with a lower prevalence of heart diseases (Table 2).

Table 3 presents the models additionally adjusted for BMI, showing that the overall pattern of associations remained consistent with those observed in Table 3. The inverse associations of processed plant-based diets with hypertension, hypercholesterolemia, and diabetes persisted after BMI adjustment, indicating effects beyond body weight. Associations for whole plant-based diets and animal-based diets also showed minimal changes. These findings suggest that BMI only partially accounts for the observed relationships, while other mechanisms are likely involved.

To confirm our findings on the 2019 survey, we performed the same univariate and multivariate analysis with the 2013 survey (Tables S3 and S4). Findings following the same pattern were observed in the 2013 survey. A high whole plant-based dietary pattern was associated with a non-significant lower prevalence of obesity (OR 0.83; 95% CI 0.63, 1.07 for a high plant-based dietary pattern; OR 0.87; 95% CI: 0.72, 1.06 for an intermediate whole plant-based dietary pattern) in comparison to a low whole plant-based dietary pattern (Table S4).

Additionally, a higher intake of processed plant-based foods was associated with a lower prevalence of obesity (OR 0.90; 95% CI 0.83, 0.97), but the association between animal-based dietary pattern and obesity was not significant (OR 0.98; 95% CI 0.75, 1.29). Other significant associations were a lower prevalence of hypertension (OR 0.82; 95% CI 0.76, 0.88), hypercholesterolemia (OR 0.81, 95% CI 0.74, 0.88), and diabetes (OR 0.53; 95% CI 0.48, 0.59) with a high intake of a processed plant-based dietary pattern when compared with a low intake. The animal-based pattern was associated with a lower prevalence of heart diseases when compared to a high vs. low intake (OR 0.60; 95% CI 0.40, 0.90) and an intermediate vs. low intake OR 0.77; 95% CI 0.60, 0.99) (Table 3).

### 3.3. Direct and Indirect Associations of Whole Plant-Based Diets and Metabolic Diseases

We conducted a mediation analysis to assess whether the associations between dietary patterns and cardiometabolic outcomes were explained by BMI. A high whole plant-based dietary pattern was associated with a significant total effect on hypertension (β = −0.18; 95% CI −0.31, −0.05) and hypercholesterolemia (β = −0.14; 95% CI −0.27, −0.009), largely explained by the indirect pathway through BMI (pure indirect effect β = −0.09 for hypertension and β = −0.06 for hypercholesterolemia; both *p* < 0.01). For hypercholesterolemia, we also observed a significant reference interaction (β = −0.12; 95% CI −0.24, −0.004), suggesting that the effect of diet on cholesterol levels may vary according to BMI status. For diabetes and heart disease, total effects were not significant, although indirect estimates through BMI reached significance (β = −0.05 and β = −0.03, respectively); however, these results produced inconsistent proportions (negative or greater than 100%), limiting their interpretability. Finally, for stroke, no indirect association with BMI was identified, but a borderline direct effect of the diet itself was observed (β = −0.34; 95% CI −0.69, 0.009), suggesting potential mechanisms independent of body weight (Table 4).

We also conducted the mediation analysis in the 2013 survey (Table S5). Similar to the 2019 survey, we found a significant negative pure indirect effect of BMI on the association between a high-plant diet and stroke. However, no significant pure indirect effects for heart diseases, and no overall effect and controlled direct effects for hypertension were observed. The size, direction, and significance of other effects remained similar between the two waves. Especially, significant negative pure indirect effects of BMI on the relationship between a high-plant diet and hypertension, hypercholesterolemia, and diabetes were still observed in the wave of 2013, and the proportion of pure indirect effects to the total effect in different waves was similar, ranging from 0–50% for high cholesterol, 50–60% for hypercholesterolemia, and 140–170% for diabetes.

## 4. Discussion

In this representative survey of the Brazilian population, a plant-based dietary pattern was associated with a lower prevalence of obesity and hypercholesterolemia. Although there are only a few randomized controlled trials (RCTs) testing the effect of plant-based diets, including lower amounts of animal protein-derived foods on obesity and hyperlipidemia [22,23], studies using the plant-based score were conducted and demonstrated the favorable effects of a plant-rich diet against excessive weight and other diseases [24].

In the United States, a study including three ongoing prospective cohorts involving 126,982 adults found that higher adherence to a plant-based diet, assessed through a plant-based diet index, was associated with lower weight gain over 4 years [25]. While a whole plant-based diet may have a protective effect against obesity and hyperlipidemia based on our results, we did not find any significant associations between whole plant-based dietary patterns in the Brazilian adult population and the prevalence of hypertension, diabetes, heart disease, and stroke. Whereas our findings are directly in line with previous studies that analyzed the relationship between this dietary pattern with obesity and hypercholesterolemia, they are in contrast with a previous study that analyzed the relationship between a plant-based dietary pattern with diabetes, hypertension, and heart diseases [26,27]. In a follow-up Swedish study of 24.3 years, participants who adhered closely to the Eat-Lancet plant-based diet had an 18% lower risk of developing type 2 diabetes mellitus (T2DM) compared to those with the lowest adherence to the diet [28].

We also analyzed the relationship between following a processed plant-based pattern and the prevalence of cardiometabolic diseases. Surprisingly, those who reported having followed a processed plant-based dietary pattern had a lower prevalence of obesity, hypertension, hypercholesterolemia, and diabetes mellitus. In contrast, an analysis of prospective cohort studies involving US health professionals, the consumption of juices, sweetened beverages, refined grains, potatoes, and sweets/desserts, has been linked to a 16% higher risk of T2DM [29]. Additionally, in another meta-analysis, a greater adherence to a plant-based dietary pattern led to a 23% reduction in the risk of T2DM. The effect was greater in those who follow a predominant healthful plant-based pattern, including fruits, vegetables, whole grains, legumes, and nuts [26]. The processed plant-based pattern in this study comprises plant-sourced refined/processed items and does not represent what is proposed as a healthful plant-based diet; observed inverse associations should be interpreted cautiously, given possible misclassification, reverse causation (change in diet after diagnosis), and residual confounding. In the context of global evidence, our findings require caution. Meta-analytic and cohort data indicate that only healthy plant-based indices are consistently associated with lower cardiometabolic risk, while unhealthy/processed plant-based indices (uPDI) are linked to higher risk—for example, the Rotterdam Study with an updated meta-analysis for CVD and dose–response evidence for type 2 diabetes [5]. Furthermore, greater exposure to ultra-processed foods shows robust links with adverse outcomes in comprehensive/meta-analytic studies and in specific cardiometabolic syntheses for CVD, CHD, and stroke [30].

The difference between our findings and those found in these studies might be attributed to different factors. It is possible that individuals diagnosed with certain NCDs are more likely to change their eating patterns, thereby influencing the observed associations. This potential reverse causality has been identified and discussed before and may be related to our findings [31]. However, even if the consumption of processed foods is not advised, some studies have also demonstrated that a dietary pattern that combines a predominant intake of plant-based foods, including refined (overall plant-based dietary index) and limited in animal-based foods, may result in more favorable outcomes [26,32].

In our study, a higher consumption of animal-based foods was not associated with obesity, hypertension, hypercholesterolemia, or diabetes when compared to a lower one. Although the animal-based pattern was associated with a lower prevalence of heart disease (OR 0.60; 95% CI 0.40–0.90), the upper bound of the confidence interval approaches the null value (OR = 1.0), indicating limited precision and compatibility with a small—or even near-null—association; this result should therefore be interpreted cautiously. The impact of the consumption of animal-based food on cardiometabolic health is controversial. While recent recommendations from the American Heart Association [33], take into account that replacing energy intake of saturated fatty acids with the intake of polyunsaturated fatty acids (PUFAs) or monounsaturated fatty acids (MUFAs) was associated with a lower risk of coronary heart disease, protection against weight gain, and the development of diabetes [34]. A recent analysis and a guideline do not recommend against the consumption of processed and unprocessed beef as a result of a null to low impact on the risk of cardiovascular disease demonstrated in different systematic reviews [11,35]. As we assessed a weekly food intake to define the animal-based dietary pattern, the amount of saturated fat per day could follow the recommendations of the AHA guidelines and may explain our results. However, a recent meta-analysis including 4.46 million participants reported a positive association between red meat consumption (both processed and unprocessed) and cardiovascular disease as well as type 2 diabetes [12]. Consistent with this, a recent large cohort analysis from the Million Veteran Program reported that participants in the highest category of total red meat intake had an 18% higher risk of cardiovascular disease compared to those in the lowest category [36]. These findings suggest that adverse effects of meat consumption—particularly at higher intakes or within less healthy dietary contexts—cannot be dismissed.

Our result might also be attributed to factors such as the composition of the diet in Brazil. The fatty acid content of different meats, such as beef, pork, and chicken, might be influenced by a wide variety of factors, including climate, animal breed and breeding, rearing conditions, and feeding [37,38]. These factors suffer regional and country-by-country influence according to cultural practices. This may be the case in Brazil, where different from most countries, 82.81% of national production is based on extensive livestock farming that consists of grazing with the occupation of large areas. Cattle are raised loose and have most of their nutrient intake from pasture [39]. Notably, grass-fed beef contained less total fat than grain-fed beef and exhibits a more favorable fatty acid lipid profile, containing fewer cholesterol-raising fatty acids. Grass-fed beef contains less total fat and saturated fatty acids than grain-fed beef, while providing higher levels of total n-3 polyunsaturated fatty acids (EPA, DPA, DHA) and a lower n-6/n-3 ratio. These characteristics, together with the enrichment in functional lipid components such as omega-3 PUFA, suggest that grass-fed beef may exert protective effects against chronic diseases, including cardiovascular disease [40]. It is important to emphasize that there is considerable scientific debate about the certainty of evidence linking meat intake with morbidity and mortality due to the absence of long-term RCTs that tested the effect of red, unprocessed, and processed meat intake aiming to reduce cardiometabolic diseases, such as obesity, T2DM, and cardiovascular disease [11,35]. All of this makes it difficult to set an upper limit for safe consumption of meat. A proper risk assessment requires considering the frequency and amount of meat intake, the method of preparation, and interactions with other compounds in the diet [35].

We also considered the consumption of fish in our analyses, which is not only a source of high-nutritional-quality protein but also a significant reserve of polyunsaturated fatty acids, especially the eicosatetraenoic (EPA-C20:5 ω-3) and docosahexaenoic (DHA-C22:6 ω-3) acids. These two fatty acids are known to present health benefits to humans because their intake has been systematically associated with a reduction in the risk of cardiovascular disease [41]. Specifically, marine and freshwater fish from Brazil have a lipid content varying according to each fish species’ behavior and breeding strategies. Species found in Brazil present great amounts of beneficial LC-PUFA content, especially EPA and DHA, being considered sources of these fatty acids. In addition, the nutritional quality indexes as well as the n-6/n/3 ratio of a variety of species indicated that their consumption could offer several benefits for human health, mostly associated with a decrease in the rates of cardiovascular disease and may explain, in part, the association of animal-based dietary patterns and heart disease [42].

We also performed a mediation analysis to further explore whether the associations observed could be explained by BMI. The analysis suggested that the inverse association between a high plant-based diet and hypercholesterolemia was partly mediated by BMI [43], while results for hypertension were weaker and should be interpreted with caution. For diabetes and heart disease, total effects were not significant, and the indirect estimates yielded inconsistent proportions; no indirect effect was observed for stroke. These findings are in line with the idea that adherence to a high-plant diet favors the maintenance of an adequate weight status, which in turn contributes to a lower risk of metabolic diseases. A recent systematic review and meta-analysis concluded that plant-based meat alternatives can improve cardiometabolic risk factors, including reductions in total cholesterol and LDL-C, supporting the potential role of plant-centered dietary choices in lipid management [43]. Furthermore, a study conducted in South Korea demonstrated a significant protective effect of a higher healthy plant-based diet index against T2DM among individuals with a BMI of 25 kg/m^2^ or higher, while this effect was not clearly demonstrated within the population without excessive weight [44]. Satija et al. also incorporated BMI as an independent variable in their regression model across the three prospective study populations and found a reduction in the protective effect of the plant-based diet of T2DM compared to the original model, accompanied by a lower significance [25]. Beyond BMI, other anthropometric or morphometric features may also act as mediators of the associations between dietary patterns and cardiometabolic outcomes. In this PNS sample, only self-reported height and weight were available; therefore, alternative anthropometric estimates could not be assessed, and their mediation effects may not be defined.

The analysis of data from the survey waves in 2013 and 2019 revealed a consistent prevalence of metabolic diseases, indicating the enduring impact of certain dietary factors on these conditions over time. Specifically, the regression analysis highlighted a low likelihood of metabolic diseases in the high-processed plant-based diet population, suggesting potential protective effects against these conditions. Further investigation into the specific components contributing to this effect could aid in the development of targeted dietary interventions. Additionally, the study found negative pure indirect effects on metabolic diseases in the high whole plant-based diet group, mediated by lower BMI categories, indicating a reduced risk of metabolic diseases for individuals adhering to this dietary pattern. Understanding the mechanisms linking a whole plant-based diet to lower BMI and disease risk could offer valuable insights. Proper consideration of confounding variables and replication in diverse populations would strengthen the findings, informing evidence-based dietary guidelines and public health interventions to combat metabolic diseases and improve population health.

This study offers several strengths. Firstly, we utilized Brazilian representative nationwide data, which boasts representative sampling methods and detailed questions, effectively capturing the country’s overall situation. Secondly, by appropriately reviewing research results based on PNS 2013, we validate findings based on PNS 2019, enriching the informativeness of similar results. Additionally, we integrated the frequency of consuming various foods into three dietary patterns and objectively evaluated plant-based diets using the concepts of “whole plant” and “processed plant” instead of the conventional “unhealthy” or “ultra-processed” labels. Our approach addresses the limitations of existing research and employs diverse analysis methods, such as stratification and mediation analysis, to fully elucidate the relationship between dietary patterns and various health outcomes. Our study presents evidence from Brazil and developing countries for research in this field of nutrition and epidemiology.

While this study has yielded significant associations, it is essential to acknowledge some limitations. The cross-sectional design of the study hinders the establishment of causality, allowing the identification of an association between dietary patterns and cardiometabolic diseases. Additionally, both exposures and outcomes were self-reported, including dietary intake, height, and weight (to derive BMI), and physician-diagnosed conditions. Self-report introduces measurement error and misclassification: reporting error in anthropometrics can lead to BMI misclassification that may attenuate associations, whereas imperfect recall or awareness of diagnoses (e.g., diabetes, hypercholesterolemia) can result in under-ascertainment and bias in uncertain directions. We lacked validation/biomarker data to quantify these errors; therefore, findings should be interpreted with appropriate caution. The assessment of food intake was limited, lacking a more detailed range of foods consumed by participants, as the data from PNS2019 provided information on the frequency of food consumption without other crucial details, such as weight and daily energy intake. To deepen our understanding of the observed relationships and potential long-term effects of different dietary patterns on health outcomes, further studies exploring underlying mechanisms and the development of clinical trials are warranted.

The differences between crude and adjusted odds ratios suggest relevant confounding by sociodemographic, behavioral, and clinical factors. For some outcomes (e.g., diabetes in Table S2), the opposite direction of crude vs. adjusted results may also reflect reverse causation (diet change after diagnosis) and misclassification from self-reported diagnoses. We, therefore, took into account, lastly, the definitive direction of the results based on the adjusted models. We performed the interpretation of adjusted estimates and their precision. Reverse causation cannot be ruled out, as the diagnosis of the disease may change dietary habits, resulting in changes in the relationship between dietary patterns and the prevalence of diseases attributed to those habits. Individuals with existing NCDs may have modified their diets after diagnosis (reverse causation). The survey does not capture the timing of diagnosis or dietary change, precluding a formal assessment of directionality.

## 5. Conclusions

In this analysis, greater adherence to a whole-food, plant-based pattern was associated with a lower prevalence of obesity and hypercholesterolemia, while a processed, plant-based pattern showed inverse associations with obesity, hypertension, hypercholesterolemia, and diabetes; a higher intake of animal-based foods was associated with a lower prevalence of heart disease. These differences may reflect how foods coexist and are grouped within each pattern, rather than a simple plant-versus-animal dichotomy. Because this is a cross-sectional study with self-reported exposures and outcomes, causality cannot be inferred. Furthermore, the observed associations may have been influenced by Brazilian consumption patterns, which may affect generalizability. Given the reliance on self-reported diet, anthropometrics, and diagnoses, the observed associations should be interpreted cautiously. Cohort studies and RCT in Brazil should test whether these associations between plant or animal-based foods persist over time and clarify the effects of components and substitution levels within these dietary patterns.

## Figures and Tables

**Table 1:** Dietary patterns classification according to weekly consumption of food items/groups.

Dietary Patterns	Food Items/ Food Group	Low Consumption	Intermediate Consumption	High Consumption
Whole Plant-based	beans	<5×	Has at least one proposed high consumption but not all	≥5×
	vegetables	<7×		7×
	fruit juice	<2×		≥2×
	fruit	<7×		7×
Processed Plant-based	juice	0	No intermediate consumption	≥1
	soda	0		
	cookies/sweets	0		
	snacks/prepared meals	0		
Animal-based	red meat	≤2×	Does not meet all high consumption or all low consumption requirements	>2×
	poultry	<3×		≥3×
	fish	<2×		≥2×
	milk	<7×		7×

Consumption refers to “1×” to “7×” refers to times per week. Source: Authors’ analysis of data from the 2019 Brazilian National Health Survey (Pesquisa Nacional de Saúde, PNS) [19]. Consumption cutoffs were defined using the distribution of intake based on dietary recommendations [20,21].

**Table 2:** Fully adjusted logistic regression models of health outcomes according to dietary patterns.

		Whole Plant-Based		Processed Plant-Based	Animal-Based	
		OR (95% CI) *p*-Value		OR (95% CI) *p*-Value	OR (95% CI) *p*-Value	
Outcome	N	High vs. Low	Intermediate vs. Low	High vs. Low	High vs. Low	Intermediate vs. Low
Obesity		0.64	0.77	0.90	0.98	0.97
	62,445	(0.54, 0.75)	(0.68, 0.88)	(0.83, 0.97)	(0.75, 1.29)	(0.83, 1.15)
		<0.001	<0.001	0.007	0.902	0.744
Hypertension		0.89	0.93	0.82	0.85	0.97
	61,466	(0.75, 1.06)	(0.81, 1.07)	(0.76, 0.88)	(0.65, 1.11)	(0.80, 1.17)
		0.184	0.308	<0.001	0.234	0.724
Hypercholesterolemia		0.69	0.87	0.81	0.92	1.028
	57,870	(0.56, 0.85)	(0.73, 1.03)	(0.74, 0.88)	(0.70, 1.22)	(0.85, 1.24)
		<0.001	0.115	<0.001	0.566	0.771
Diabetes		0.88	1.00	0.53	1.13	1.03
	58,572	(0.680, 1.14)	(0.80, 1.25)	(0.48, 0.59)	(0.79, 1.60)	(0.80, 1.32)
		0.345	0.981	<0.001	0.512	0.829
Stroke		0.76	0.72	0.98	1.07	0.76
	62,445	(0.49, 1.20)	(0.50, 1.03)	(0.81, 1.17)	(0.55, 2.09)	(0.52, 1.11)
		0.239	0.072	0.795	0.841	0.161
Heart Disease		1.03	1.06	0.89	0.60	0.77
	62,445	(0.77, 1.38)	(0.83, 1.36)	(0.78, 1.02)	(0.40, 0.90)	(0.60, 0.99)
		0.834	0.634	0.088	0.013	0.045

Fully adjusted logistic regression models of health outcomes (obesity, hypertension, hypercholesterolemia, diabetes, stroke, heart disease) according to dietary patterns in Brazilian adults (PNS 2019). Models adjusted for age, sex assigned at birth, race, marital status, household income, education, urban/rural residence, region, physical activity, smoking, alcohol intake, and dietary patterns. Results expressed as odds ratios (OR) with 95% confidence intervals (CI).

**Table 3:** Fully adjusted logistic regressions of health outcomes according to dietary patterns with BMI.

		Whole Plant-Based		Processed Plant-Based	Animal-Based	
		OR (95% CI) *p*-Value		OR (95% CI) *p*-Value	OR (95% CI) *p*-Value	
Outcome	N	High vs. Low	Intermediate vs. Low	High vs. Low	High vs. Low	Intermediate vs. Low
Hypertension		0.98	0.98	0.82	0.83	0.94
	61,466	(0.82, 1.16)	(0.85, 1.14)	(0.76, 0.89)	(0.63, 1.09)	(0.78, 1.14)
		0.808	0.818	<0.001	0.171	0.559
Hypercholesterolemia		0.72	0.89	0.81	0.91	1.02
	57,870	(0.59, 0.89)	(0.75, 1.06)	(0.74, 0.88)	(0.69, 1.21)	(0.84, 1.23)
		0.002	0.186	<0.001	0.519	0.855
Diabetes		0.95	1.03	0.53	1.09	1.01
	58,572	(0.73, 1.23)	(0.83, 1.30)	(0.48, 0.59)	(0.77, 1.55)	(0.79, 1.30)
		0.693	0.767	<0.001	0.629	0.930
Stroke		0.79	0.73	0.98	1.07	0.77
	62,445	(0.50, 1.24)	(0.51, 1.05)	(0.82, 1.18)	(0.55, 2.10)	(0.52, 1.12)
		0.305	0.091	0.846	0.839	0.172
Heart Disease		1.070	1.09	0.90	0.59	0.77
	62,445	(0.80, 1.43)	(0.85, 1.39)	(0.78, 1.03)	(0.40, 0.89)	(0.60, 0.99)
		0.636	0.519	0.113	0.012	0.045

Fully adjusted logistic regression models of health outcomes (obesity, hypertension, hypercholesterolemia, diabetes, stroke, heart disease) according to dietary patterns, additionally adjusted for BMI (PNS 2019). Same covariates as Table 3 plus BMI. Results expressed as odds ratios (OR) with 95% confidence intervals (CI).

**Table 4:** Mediation analysis for 2019.

	Coefficient	95% CI	*p*-Value	%	N
**Hypertension**	−0.18	−0.31, −0.05	0.007	100%	6920
Controlled direct association	0.001	−0.09, 0.09	0.982	−1%	
Reference interaction	−0.10	−0.23, 0.02	0.101	59%	
Mediated interaction	0.02	−0.004, 0.04	0.118	−10%	
Pure indirect association	−0.09	−0.13, −0.05	0	51%	
**Hypercholesterolemia**	−0.14	−0.27, −0.009	0.036	100%	6579
Controlled direct association	0.02	−0.13, 0.16	0.822	−11%	
Reference interaction	−0.12	−0.24, −0.004	0.043	86%	
Mediated interaction	0.02	−0.0007, 0.04	0.058	−15%	
Pure indirect association	−0.06	−0.08, −0.03	0	41%	
**Diabetes**	−0.03	−0.24, 0.17	0.76	100%	6621
Controlled direct association	−0.06	−0.25, 0.13	0.514	201%	
Reference interaction	0.10	−0.08, 0.28	0.274	−321%	
Mediated interaction	−0.01	−0.05, 0.01	0.284	55%	
Pure indirect association	−0.05	−0.08, −0.02	0	165%	
**Heart diseases**	0.04	−0.21, 0.29	0.75	100%	7020
Controlled direct association	0.11	−0.17, 0.38	0.455	260%	
Reference interaction	−0.04	−0.22, 0.15	0.702	−90%	
Mediated interaction	0.01	−0.02, 0.04	0.703	15%	
Pure indirect association	−0.03	−0.06, −0.007	0.012	−84%	
**Stroke**	−0.24	−0.54, 0.05	0.101	100%	7020
Controlled direct association	−0.34	−0.69, 0.009	0.056	139%	
Reference interaction	0.12	−0.14, 0.38	0.368	−48%	
Mediated interaction	−0.02	−0.06, 0.02	0.376	8%	
Pure indirect association	−0.004	−0.04, 0.03	0.799	2%	

Mediation analysis (four-way decomposition) of the association between a high whole plant-based dietary pattern and cardiometabolic outcomes in Brazilian adults (PNS 2019). BMI was tested as a mediator. Coefficients, 95% confidence intervals (CI), *p*-values, and proportion mediated (%) are shown. N corresponds to sample size per outcome.

## Data Availability

The data that support the findings of this study are publicly available from the Instituto Brasileiro de Geografia e Estatística (IBGE) *as part of the* Brazilian National Health Survey (Pesquisa Nacional de Saúde—PNS) *at:* https://www.ibge.gov.br/estatisticas/sociais/saude/9160-pesquisa-nacional-de-saude.html?=&t=microdados (accessed on 15 August 2025).

## Supplementary Materials

The following supporting information can be downloaded at: https://www.mdpi.com/article/10.3390/nu17213448/s1, Table S1: Sample characteristics (2019); Table S2: Unadjusted logistic regression of health outcomes according to dietary patterns 2019; Table S3 Unadjusted logistic regression of health outcomes according to dietary patterns 2013; Table S4: Adjusted logistic regression of health outcomes according to dietary patterns 2013; Table S5: Mediation analysis for 2013.

## Abbreviations

The following abbreviations are used in this manuscript:

NCDs	Noncommunicable Diseases
BMI	Body Mass Index
PNS	Brazilian National Health Survey (Pesquisa Nacional de Saúde)
IBGE	Brazilian Institute of Geography and Statistics (Instituto Brasileiro de Geografia e Estatística)
FFQ	Food Frequency Questionnaire
RCT	Randomized Controlled Trial
PUFA	Polyunsaturated Fatty Acids
MUFA	Monounsaturated Fatty Acids
EPA	Eicosapentaenoic Acid
DHA	Docosahexaenoic Acid
LC-PUFA	Long-Chain Polyunsaturated Fatty Acids
AHA	American Heart Association
T2DM	Type 2 Diabetes Mellitus
DAG	Directed Acyclic Graph
STROBE	Strengthening the Reporting of Observational Studies in Epidemiology
FIPE/HCPA	Research Incentive Fund—Hospital de Clínicas de Porto Alegre
CNPq	National Council for Scientific and Technological Development (Conselho Nacional de Desenvolvimento Científico e Tecnológico)
