# Supplementary material for: Plant and Animal-Based Dietary Patterns and Cardiometabolic Diseases in the Brazilian Population: Cross-Sectional Analysis of the Brazilian National Health Survey
Source: Nutrients. 2025 Oct 31;17(21):3448. doi: 10.3390/nu17213448 (PMC12608340; doi:10.3390/nu17213448)
Supplement: Supplementary file 1 [file nutrients-17-03448-s001.zip › nutrients-3867540-supplementary.pdf]

Supplementary Materials

Table S1. Sample characteristics (2019)

Variables			Low whole plant-based consumption	Intermediate whole plant-based consumption	High whole plant-based consumption	p-value
	N	Total	7,553	72,179	7,946	
Age, mean(sd)	87,678	47.36 (17.09)	44.15 (17.18)	47.18 (17.02)	52.05 (16.72)	0.042
**Sex**	87,678					
Female		46,016 (52.48)	4,041(53.50)	37,247 (51.60)	4,728 (59.50)	<0.001
**Geographical region**	87,678					
North		16,772 (19.07)	2,409 (31.89)	13,465 (18.66)	848 (10.67)	<0.001
Northeast		30,415 (34.69)	2,072 (27.43)	25,528 (35.37)	2,815 (35.43)	
Southeast		19,285 (22.00)	1,169 (15.48)	15,802 (21.89)	2,314 (29.12)	
South		11,186 (12.78)	1,279 (16.93)	9,045 (12.53)	862 (10.85)	
Midwest		10,070 (11.49)	624 (8.26)	8,339 (11.55)	1,107 (13.93)	
**Type of residence**	87,678					
Urban		67,567 (77.06)	5,652 (74.83)	55,221 (76.51)	6,694 (84.24)	<0.001
Rural		20,111 (22.94)	1,901 (25.17)	16,958 (23.49)	1,252 (15.76)	
**Skin Color/Race**	87,669					
White		32,161 (36.68)	2,580 (34.17)	26,137 (36.21)	3,444 (43.34)	<0.001
Black		10,017 (11.43)	848 (11.23)	8,324 (11.53)	845 (10.63)	
Mixed		44,169 (50.38)	3,993 (52.88)	36,617 (50.74)	3,559 (44.79)	
Yellow		660 (0.75)	50 (0.66)	557 (0.77)	53 (0.67)	
Indigenous		662 (0.76)	80 (1.06)	537 (0.74)	45 (0.57)	
**Marital Status**	87,678					<0.001
Married		34,798 (39.69)	2,236 (29.60)	28,851 (39.97)	3,711 (46.70)	
**2. Socioeconomic status**						
**Household income, mean (sd)**	87,657	3890.74 (6264.49)	3213.16 (5283.82)	3841.09 (6264.96)	4985.97 (6950.38)	<0.001
**Income (per capita household income range)**	87,657					
Less than 2 minimum wages		69,725 (79.54)	6,316 (83.64)	57,789 (80.08)	5,620 (70.75)	<0.001
2 to 3 minimum wages		7,466 (8.52)	542 (7.18)	6,003 (8.32)	921 (11.60)	
Higher than 3 minimum wages		10,466 (11.94)	693 (9.18)	8,371 (11.60)	1,402 (17.65)	
**Education**	75,782					
Illiterate and Incomplete elementary school, elementary		34,680 (45.76)	2,955 (47.90)	28,847 (46.16)	2,878 (40.40)	<0.001
High School		26,908 (35.51)	2,162 (35.05)	22,163 (35.47)	2,583 (36.26)	
University		14,194 (18.73)	1,052 (17.05)	11,479 (18.37)	1,663 (23.34)	
**3. HEALTH**						
BMI, mean(sd)	87,678	26.50 (4.90)	26.56 (5.10)	26.49 (4.78)	26.46 (4.52)	<0.001
**Smoking status**	76,454					

Never smoked		52,636 (68.85)	4,223 (67.45)	43,269 (68.81)	5,144 (70.32)	0.001
Current or ex-smokers		23,818 (31.15)	2,038 (32.55)	19,609 (31.19)	2,171 (29.68)	
**Alcohol consumption**	23,997					
One or more than once per monthly		11,215 (46.74)	1,036 (48.89)	9,362 (46.77)	817 (43.90)	0.007
**Physical Activity**						
≥ 150 min/week	87,678	28,802 (32.85)	1,881 (24.90)	23,429 (32.46)	3,492 (43.95)	<0.001
< 150 min/week		58,876 (67.15)	5,672 (75.10)	48,750 (67.54)	4,454 (56.05)	
**4. OUTCOMES**						
Obesity	87,678	18,026 (20.56)	1,692 (22.40)	14,768 (20.46)	1,566 (19.71)	<0.001
Diabetes	81,524	7,076 (8.68)	486 (7.32)	5,770 (8.59)	820 (10.61)	<0.001
Hypertension	86,016	22,496 (26.15)	1,660 (22.81)	18,340 (25.89)	2,496 (31.61)	<0.001
Hypercholesterolemia	80,443	13,349 (16.59)	995 (15.25)	10,931 (16.51)	1,423 (18.50)	<0.001
Heart diseases (heart attack, angina, heart failure)	87,678	4,706 (5.37)	369 (4.89)	3,776 (5.23)	561 (7.06)	<0.001
Stroke	87,678	1,971 (2.25)	173 (2.29)	1,590 (2.20)	208 (2.62)	0.059
**5. CONSUMPTION OF PROCESSED PLANT-BASED AND ANIMAL-BASED FOOD**	87,678					
**Processed plant-based food**						
Low processed plant-based consumption		22,551 (25.72)	1,670 (22.11)	18,201 (25.22)	2,680 (33.73)	
High processed plant-based consumption		65,127 (74.28)	5,883 (77.89)	53,978 (74.78)	5,266 (66.27)	<0.001
**Animal-based food**						
Low animal-based consumption		4,950 (5.65)	754 (9.98)	3,919 (5.43)	277 (3.49)	<0.001
Intermediated animal-based consumption		80,368 (91.66)	6,688 (88.55)	66,387 (91.98)	7,293 (91.78)	
High animal-based consumption		2,360 (2.69)	111 (1.47)	1,873 (2.59)	376 (4.73)	

Sociodemographic, lifestyle, and health characteristics of Brazilian adults according to whole plant-based dietary pattern intake (low, intermediate, high) in the PNS 2019 (n = 87,678). Continuous variables are presented as mean (SD); categorical variables as n (%). p-values obtained from chi-square tests or ANOVA. Abbreviations: SD, standard deviation; BMI, body mass index**.**

Table S2

Table S2. Unadjusted logistic regression of health outcomes according to dietary patterns 2019.

	**Whole Plant-based**	**Refined Plant-based**	**Animal-based**
	OR (95% CI) P-value	OR (95% CI) P-value	OR (95% CI) P-value

Outcome	N	High vs Low	Intermediate vs Low	High vs Low	High vs Low	Intermediate vs Low
Obesity	87,678	0.74<br>(0.65, 0.86)<br><0.001	0.82<br>(0.73, 0.91)<br><0.001	0.89<br>(0.83, 0.95)<br><0.001	0.99<br>(0.77, 1.25)<br>0.92	0.97<br>(0.84, 1.11)<br>0.62
Hypertension	86,016	1.59<br>(1.40, 1.81)<br><0.001	1.28<br>(1.16, 1.42)<br><0.001	0.49<br>(0.46, 0.52)<br><0.001	0.89<br>(0.73, 1.10)<br>0.29	0.90<br>(0.79, 1.02)<br>0.10
Hypercholesterolemia	80,443	1.02<br>(0.86, 1.19)<br>0.85	1.01<br>(0.88, 1.15)<br>0.13	0.58<br>(0.55, 0.62)<br><0.001	0.95<br>(0.74, 1.21)<br>0.66	0.94<br>(0.81, 1.10)<br>0.46
Diabetes	81,524	1.36<br>(1.10, 1.68)<br>0.005	1.24<br>(1.04, 1.49)<br>0.017	0.35<br>(0.32, 0.38)<br><0.001	0.96<br>(0.71, 1.29)<br>0.79	0.98<br>(0.80, 120)<br>0.87
Stroke	87,678	1.32<br>(0.89, 1.97)<br>0.17	1.00<br>(0.77, 1.31)<br>0.99	0.53<br>(0.45, 0.62)<br><0.001	1.32<br>(0.78, 2.22)<br>0.30	0.95<br>(0.69, 1.30)<br>0.73
Heart Disease	87,678	1.47<br>(1.17, 1.84)<br>0.001	1.19<br>(0.99, 1.43)<br>0.071	0.63<br>(0.56, 0.70)<br><0.001	0.58<br>(0.41, 0.82)<br>0.002	0.71<br>(0.58, 0.88)<br>0.001

Unadjusted logistic regression models of health outcomes according to dietary patterns in Brazilian adults (PNS 2019). Results expressed as odds ratios (OR) with 95% confidence intervals (CI).

Table S3.

Table S3. Unadjusted logistic regression of health outcomes according to dietary patterns 2013

		Whole Plant-based		Refined Plant-based	Animal-based	
		OR (95% CI) P-value		OR (95% CI) P-value	OR (95% CI) P-value	
**Outcome**	**N**	**High vs Low**	**Intermediate vs Low**	**High vs Low**	**High vs Low**	**Intermediate vs Low**
Obesity	43,180	0.92 (0.73, 1.14) 0.44	0.90 (0.76, 1.05) 0.18	0.82 (0.75, 0.91) <0.001	1.25 (0.89, 1.75) 0.19	1.41 (1.08, 1.85) 0.012
Hypertension	42,227	1.73 (1.38, 2.17) <0.001	1.23 (1.04, 1.45) 0.015	0.64 (0.58, 0.70) <0.001	1.22 (0.89, 1.66) 0.21	1.08 (0.86, 1.36) 0.48
Hypercholesterolemia	37,907	1.12 (0.86, 1.45) 0.41	0.90 (0.74, 1.08) 0.26	0.77 (0.69, 0.85) <0.001	0.74 (0.50, 1.09) 0.13	0.85 (0.63, 1.15) 0.30
Diabetes	38,970	1.81 (1.23, 2.65) 0.002	1.29 (0.95, 1.74) 0.10	0.41 (0.35, 0.47) <0.001	0.94 (0.52, 1.72) 0.85	0.98 (0.64, 1.51) 0.94
Stroke	43,180	1.76 (0.94, 3.32) 0.079	1.00 (0.62, 1.62) 0.99	0.48 (0.35, 0.65) <0.001	0.92 (0.27, 3.11) 0.89	0.88 (0.32, 2.44) 0.81
Heart Disease	43,180	1.58 (1.01, 2.47) 0.043	0.92 (0.67, 1.28) 0.64	0.75 (0.62, 0.89) 0.001	0.71 (0.39, 1.31) 0.27	0.54 (0.37, 0.79) 0.002

Unadjusted logistic regression models of health outcomes according to dietary patterns in Brazilian adults (PNS 2013). Results expressed as odds ratios (OR) with 95% confidence intervals (CI).

Table S4.

.

Table S4. Adjusted logistic regression of health outcomes according to dietary patterns 2013.

		Whole Plant-based		Refined Plant-based	Animal-based	
		OR (95% CI) P-value		OR (95% CI) P-value	OR (95% CI) P-value	
**Outcome**	**N**	**High vs Low**	**Intermediate vs Low**	**High vs Low**	**High vs Low**	**Intermediate vs Low**
Obesity	**43180**	0.83<br>(0.63, 1.07)<br>0.15	0.87<br>(0.72, 1.06)<br>0.16	0.81<br>(0.73, 0.90)<br><0.001	1.09<br>(0.73, 1.63)<br>0.66	1.38<br>(1.00, 1.92<br>0.053
Hypertension	**42227**	0.94<br>(0.70, 1.27)<br>0.70	0.90<br>(0.71, 1.13)<br>0.36	0.85<br>(0.76, 0.95)<br>0.004	1.10<br>(0.76, 1.58)<br>0.61	1.16<br>(0.89, 1.52)<br>0.28
Hypercholesterolemia	**37907**	0.78<br>(0.57, 1.06)<br>0.11	0.76<br>(0.60, 0.96)<br>0.020	0.86<br>(0.75, 0.98)<br>0.024	0.56<br>(0.36, 0.87)<br>0.010	0.73<br>(0.52, 1.02)<br>0.064
Diabetes	**38970**	0.93<br>(0.58, 1.47)<br>0.75	0.93<br>(0.65, 1.34)<br>0.69	0.45<br>(0.38, 0.53)<br><0.001	0.80<br>(0.39, 1.62)<br>0.54	1.00<br>(0.64, 1.54)<br>0.99
Stroke	**43180**	1.14<br>(0.57, 2.28)<br>0.71	0.75<br>(0.45, 1.27)<br>0.29	0.81<br>(0.58, 1.14)<br>0.24	0.94<br>(0.23, 3.82)<br>0.93	0.82<br>(0.24, 2.78)<br>0.75
Heart Disease	**43180**	0.89<br>(0.53, 1.51)<br>0.67	0.72<br>(0.47, 1.08)<br>0.12	0.97<br>(0.79, 1.19)<br>0.76	0.68<br>(0.31, 1.49)<br>0.34	0.60<br>(0.36, 0.99)<br>0.046

Fully adjusted logistic regression models of health outcomes according to dietary patterns in Brazilian adults (PNS 2013). Models adjusted for the same covariates as Table 3. Results expressed as odds ratios (OR) with 95% confidence intervals (CI)

Table S5.

Table S5. Mediation analysis for 2013

	Coefficient	C.I	P-Value	%	N
**Hypertension**	-0.02	-0.28, 0.23	0.856	100%	
Controlled direct association	0.07	-0.10, 0.24	0.412	-298%	
Reference interaction	0.01	-0.23, 0.26	0.923	-50%	2,504
Mediated interaction	-0.002	-0.05, 0.04	0.923	9%	
Pure indirect association	-0.10	-0.17, -0.04	0.001	439%	
**Hypercholesterolemia**	-0.34	-0.52, -0.16	<0.001	100%	
Controlled direct association	-0.13	-0.34, 0.09	0.253	37%	
Reference interaction	-0.19	-0.36, -0.02	0.033	56%	2,278
Mediated interaction	0.04	-0.002, 0.08	0.061	-12%	
Pure indirect association	-0.06	-0.11, -0.02	0.003	19%	
**Diabetes**	-0.17	-0.51, 0.17	0.322	100%	
Controlled direct association	0.12	-0.22, 0.45	0.495	-68%	
Reference interaction	-0.23	-0.51, 0.06	0.122	134%	2,346
Mediated interaction	0.05	-0.02, 0.11	0.151	-27%	
Pure indirect association	-0.10	-0.17, -0.04	0.002	62%	
**Heart diseases**	-0.16	-0.54, 0.22	0.415	100%	
Controlled direct association	-0.07	-0.49, 0.36	0.761	42%	
Reference interaction	-0.05	-0.37, 0.27	0.759	32%	2,561
Mediated interaction	0.009	-0.05, 0.07	0.760	-6%	
Pure indirect association	-0.05	-0.10, 0.001	0.056	32%	
**Stroke**	-0.11	-0.72, 0.50	0.727	100%	
Controlled direct association	0.32	-0.39, 1.02	0.378	-290%	
Reference interaction	-0.41	-0.85, 0.03	0.065	378%	2,561
Mediated interaction	0.07	-0.02, 0.17	0.106	-68%	
Pure indirect association	-0.09	-0.17, -0.01	0.031	80%	

Mediation analysis (four-way decomposition) of the association between a high whole plant-based dietary pattern and cardiometabolic outcomes in Brazilian adults (PNS 2013). BMI was tested as a mediator. Coefficients, 95% confidence intervals (CI), p-values, and proportion mediated (%) are shown.
